# Adaptive Response in Animals Exposed to Non-Ionizing Radiofrequency Fields: Some Underlying Mechanisms

**DOI:** 10.3390/ijerph110404441

**Published:** 2014-04-22

**Authors:** Yi Cao, Jian Tong

**Affiliations:** School of Public Health, Medical College of Soochow University, Suzhou 215123, China; E-Mail: tongjian@suda.edu.cn

**Keywords:** radiofrequency fields, adaptive response, mechanisms, mice

## Abstract

During the last few years, our research group has been investigating the phenomenon of adaptive response in animals exposed to non-ionizing radiofrequency fields. The results from several separate studies indicated a significant increase in survival, decreases in genetic damage as well as oxidative damage and, alterations in several cellular processes in mice pre-exposed to radiofrequency fields and subsequently subjected to sub-lethal or lethal doses of γ-radiation or injected with bleomycin, a radiomimetic chemical mutagen. These observations indicated the induction of adaptive response providing the animals the ability to resist subsequent damage. Similar studies conducted by independent researchers in mice and rats have supported our observation on increased survival. In this paper, we have presented a brief review of all of our own and other independent investigations on radiofrequency fields-induced adaptive response and some underlying mechanisms discussed.

## 1. Introduction

With the introduction of wireless communication systems, mobile phones have become the greatest source of public exposure to non-ionizing electromagnetic radiofrequency fields (RF). Several investigators have examined the biological effects of RF exposure in human and animal cells both in *in vitro* and in *in vivo* investigations. Recently, the International Agency for Research on Cancer has reviewed all human epidemiological investigations, carcinogenicity studies in animals and other relevant peer-reviewed scientific publications. The conclusion was that RF exposure is a possible carcinogen in the category 2B [1]. The focus of our research group has been on the examination of possible beneficial effects of whole body exposure of mice to RF. We were interested in the phenomenon of adaptive response (AR) which was originally demonstrated in bacterial cells grown in low, non-toxic dose (adaptive dose, AD) of a chemical mutagen and then treated with a high dose (challenge dose, CD) of the same mutagen. The observations were that the cells exposed to AD + CD became resistant to the damage caused by the subsequent CD suggesting the induction of AR [2]. Further *in vitro* and *in vivo* investigations in human and animal cells have also revealed cross-resistance to other mutagens and, some potential mechanisms involved in the induction of AR were proposed [3]. In this paper, we have presented a brief review of all of our own and other independent investigations on RF-induced AR and some mechanisms involved in eliciting such response discussed.

## 2. Experimental Section

### 2.1. RF Exposure

A large in-house built Gigahertz Transverse Electromagnetic (GTEM) chamber, RF signal generator and a power amplifier provided the source for continuous wave 900 MHz RF exposure of mice. The RF field inside the GTEM was probed using a field strength meter. The precise location that provided the required 12, 120 and 1200 μW/cm^2^ power density was determined. The power was continuously monitored and recorded in a computer-controlled data logging system. Small plastic box(es) containing a single restrained mouse was placed at the precise location that provided the required power density. The animals were oriented horizontally and transverse to the long axis of the GTEM chamber. The field in the chamber was horizontally polarised so that the electric (E) field was aligned with the long axis of the mice. The specific absorption rate (SAR) was calculated using finite-difference-time-domain (FDTD) analysis of a male mouse model exposed in a continuous wave, horizontally polarized, plane-wave environment at 900 MHz RF [4,5]. The calculated whole body average SAR at the measured power densities of 12, 120 and 1200 μW/cm^2^ was 5.48, 54.8 and 548 mW/kg, respectively.

### 2.2. Animals and Treatment

The experimental protocol used in all our studies was similar. Adult mice were exposed (whole body) to RF/sham for 1–4 h/day for 1–14 days (AD). Then, they were exposed to an acute, sub-lethal or lethal dose of γ-irradiation (depending on the objective of the investigation) or injected with a genotoxic dose of bleomycin (BLM), a radiomimetic chemical mutagen (CD). All mice were sacrificed later and several biological endpoints were evaluated. The observations in mice exposed to AD+CD were compared with those exposed to CD alone. The Animal Care/User Ethical Committee of Soochow University, Suzhou City, P.R. China has reviewed and approved our handling of animals and experimental protocols (Approval number A68–2011). 

## 3. Results and Discussion

In the first study, Kunming mice were exposed to RF at 120 W/cm^2^ power density for 1 hour/day for 14 days. At the end of the last RF exposure, the animals were subjected to sub-lethal dose of 5.0 Gy γ-irradiation and sacrificed after 3, 6, 9 and 12 days. Compared with the mice exposed to γ-irradiation alone, the results in mice exposed to AD + CD were as follows:

(i) Microscope slides prepared from the bone marrow and spleen tissues showed a significant and progressive decrease in damage as the time progressed after irradiation.
(ii) There were increased numbers of colony forming units (CFU-BM) in the bone marrow.
(iii) There were increased levels of colony stimulating factor (CSF) and interleukin-3 (IL-3) in the serum. These indices suggested RF-induced AR helping in faster regeneration and restoration of hematopoietic tissue damaged by subsequent γ-radiation [6].

Several separate investigations were performed further:

(1) Kunming mice were exposed to RF at 12, 120 and 1200 μW/cm^2^ power density for 1 h/day for 14 days and then subjected to lethal dose of 8.0 Gy γ-radiation. After 15 days, the numbers of surviving mice were 11, 43 and 25%, respectively (18% in mice exposed to γ-radiation alone) (Table 1). Since RF exposure at 120 μW/cm^2^ power density resulted in maximum survival advantage, another experiment was conducted at this power density and there was a significant increase in weights of spleen and thymus compared with those exposed to 8.0 Gy γ-radiation alone.

**Table 1:** Survival study in mice which were pre-exposed to 900 MHz RF with and without subsequent exposure to γ-radiation.

Group	Treatment	Power Density	γ-Radiation	# Mice Studied	# Mice Alive *	% Survival
1	γ-Radiation	-	8 Gy	28	5	18
2	900 MHz RF	12 µW/cm2	8 Gy	28	3	11
3	900 MHz RF	120 µW/cm2	8 Gy	28	12	43 **
4	900 MHz RF	1200 µW/cm2	8 Gy	28	7	25
5	Amifostine	-	8 Gy	28	8	29

Notes: *: 15 days after exposure to 8 Gy γ-radiation. **: *p* ≤ 0.05. Data from Cao *et al.* [7].
(2) The same RF exposure protocol and a sub-lethal dose of 5.0 Gy γ-radiation was used in the next set of experiments. Mice exposed to RF+5.0 Gy γ-radiation showed:
  (i) significantly increased CFU-BM,
  (ii) significantly increased expression levels of genes related to the cell cycle, viz., cyclin-D1, cyclin-E, cyclin-DK4 and cyclin-DK2 in the spleen, and
  (iii) lethally irradiated “recipient” mice injected with bone marrow cells from RF + 5.0 Gy γ-radiation exposed mice showed increased numbers of colony forming units in their spleen (CFU-S). Overall, these observations provided some mechanistic evidence for RF-induced AR in mitigating the damage induced by subsequent γ-radiation [7].

The extent of genetic damage in hematopoietic cells was examined in subsequent studies. ICR mice were exposed to RF at 120 µW/cm^2^ for 4 h/day for 1, 3, 5, 7 and 14 days. Four hours after the last RF exposure, the animals were subjected to a lower dose of 3.0 Gy γ-radiation. The results obtained from the alkaline comet assay showed a significant decrease primary DNA damage in peripheral blood leukocytes correlating with the number of RF exposures. The maximum decrease occurred after 7 days RF exposure although the decrease after 14 days was still significant compared with the mice exposed to γ-radiation alone [8]. In a similar RF and γ-radiation exposure protocol (7 days exposure only), there was a significant decrease in micronuclei (MN) in both blood and bone marrow tissues [9].

Thus, RF-induced AR was able to alleviate the genetic damage induced by subsequent γ-radiation. In a most recent investigation, mice were exposed to continuous wave 900 MHz radiofrequency fields (RF) at 120 μW/cm^2^ power density (calculated SAR 54.8 mW/kg) for 4 h/day for 7 days. Four hours after the last RF exposure, some of the animals were injected with bleomycin (BLM), a radiomimetic chemical mutagen. Compared with the animals injected with BLM alone, the results in mice exposed to RF + BLM indicated:

(i) an accelerated repair (% damage that is remaining at different repair times) of BLM-induced primary DNA damage,
(ii) significantly reduced level of oxidative damage assessed from malondialdehyde (MDA) levels, and
(iii) considerable increase in SOD, an anti-oxidant enzyme [10].

**Figure 1:**
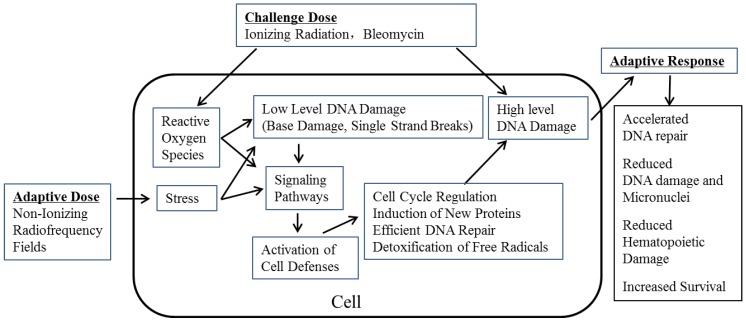
Molecular mechanisms involved in RF-induced adaptive response. Exposure of animals (and human cells) to non-ionizing radiofrequency fields, given as an adaptive dose, may generate some “stress” which may cause undetectable DNA damage. These may stimulate signal transduction pathways leading to the activation of cell defense mechanisms. The reactive oxygen species induced by ionizing radiation and BLM (the challenge dose) also play a role in the activation of cell defenses through signaling pathways. The activated cell defenses provide the cell the ability to resist high level damages induced by subsequent exposure to ionizing radiation and BLM (as challenge dose).

The same GTEM chamber was used for an *in vitro* experiment to expose human promyelocytic leukemia HL-60 cells to 900 MHz RF at 12 μW/cm^2^ power density for 1 h/day for three consecutive days (AD). The RF dosimetry was obtained using Burkhard’s formula [11]. The calculated numerical SARs was extremely low, an average 2.5 × 10^−5^ W/kg and peak 4.1 × 10^−5^ W/kg. After the last RF exposure, the cells were treated with doxorubicin (DOX, CD), a chemotherapeutic drug. A significantly increased viability, decreased apoptosis, increased mitochondrial membrane potential (MMP), decreased intracellular free Ca^2+^ and increased Ca^2+^-Mg^2+^-ATPase activity were observed in cells exposed to RF + DOX compared with those treated with DOX alone. Thus, the HL-60 cells were able to activate several cellular processes to resist the damage induced by DOX [12]. The clinical significance of these observations needs further investigations. Thus, all of these data provided some mechanistic evidence for RF-induced AR (Figure 1). 

Our observations of significant survival advantage in mice exposed to RF + γ-radiation was supported by several reports from an independent group of researchers. For RF exposure, these investigators have used an in-house built GSM mobile phone signal simulator that can be adjusted to transmit RF frequencies between 800 and 1000 MHz at 2 W power density (the details of power density measurement, monitoring and recording were not reported). In other studies, the antenna of commercially available GSM mobile phones, in talk mode, was kept at distance of 5 cm from the head of restrained animals and, the SAR provided by the mobile phone manufacturers was considered as the SAR in the exposed animals. Sprague-Dawley or Wistar rats and Balb/c mice were exposed to RF for 2–12 h/day for 3–5 days (AD). This was followed by LD_50/6_ or LD_50/30_ dose of γ-irradiation or injection with *E. coli* bacteria (CD). Several days after CD, the number of animals that were alive was recorded. The results showed a consistent pattern of significantly increased survival in animals exposed to AD + CD compared to those subjected to CD alone. Although there was insufficient description of RF dosimetry in these studies, the outcome from all of these independent reports was similar. Rats and mice which were pre-exposed to RF were able to resist the damage caused by sub-lethal and lethal doses of γ-irradiation or *E. coli* bacterial infection [13,14,15,16].

Other independent investigators also reported that human peripheral blood lymphocytes which were pre-exposed to RF were able to resist the genetic damage induced by subsequent treatment with mitomycin C, a chemotherapeutic chemical mutagen or ionizing radiation [17,18,19,20]. These data provided further support that RF pre-exposure is capable inducing AR to provide protection to human cells from subsequent damage. 

In this manuscript, we have briefly described the results obtained in our own investigations in mice exposed to RF and subsequently subjected to various doses of γ-radiation (depending on the objective of the study). We have also discussed the additional strength gained from independent investigators who reported similar RF-induced AR in mice, rats and also in human cells. However, there are several more “gaps in knowledge” in RF-induced AR other than those presented in Figure 1. A more extensive review has been published recently addressing these issues [21]. Our future investigations are directed to fill-in, at least, some of these gaps.

## 4. Conclusions

Thus far, the focus of research on the biological effects of RF exposure has been on the adverse effects. The observations in the studies reviewed above suggested that RF exposure was beneficial and able to provide resistance in animals and human cells to the damage induced by subsequent exposure to sub-lethal and lethal doses of ionizing radiation and chemical mutagens. Our data also provided some mechanistic evidence for RF-induced AR. Several other mechanisms need further examination.
